# An analysis of the crystal structure, vibrational properties, and optoelectronic behavior of NdCaFeSnO_6_

**DOI:** 10.1039/d5ra02722e

**Published:** 2025-07-04

**Authors:** Sondes Chahla, Yaovi Gagou, Mimoun El Marssi, Hanen Chaker, Rached Ben Hassen

**Affiliations:** a Laboratory of Materials and Environment for Sustainable Development (LR18ES10) ISSBAT, University of Tunis El Manar Tunis Tunisia hanen_chaker@yahoo.fr; b LPMC, Université of Picardie Jules Verne 33 rue Saint Leu, CEDEX 01 80039 Amiens France

## Abstract

The co-substitution of neodymium (Nd) and iron (Fe) into CaSnO_3_ provides, for the first time, the double perovskite-like structure NdCaFeSnO_6_. A comprehensive investigation of the structural, microstructural, vibrational (Raman), optical and electrical properties of the resulting NdCaFeSnO_6_ compound, synthesized *via* a solid-state method, is reported. Elemental analysis using Energy-Dispersive X-ray spectroscopy (EDX) confirmed the successful incorporation of Nd, Fe, Ca, Sn and O. Semi-quantitative analysis results are consistent with the intended stoichiometry. X-ray diffraction (XRD) investigation revealed a monoclinic *P*2_1_/*c* crystal structure, with a minor secondary phase. Rietveld refinement converges to satisfactory factors, indicating an equidistribution of Fe and Sn cations between the octahedral sites. Raman spectroscopy further characterized this symmetry, showing several bands associated with the bending and stretching modes of (Sn/Fe)O_6_ octahedra. Significant narrowing of the bandgap from 4.27 eV for the pristine CaSnO_3_ to 2.44 eV in NdCaFeSnO_6_ supports the presence of structural defects and oxygen vacancies within the (Nd/Ca)–(Fe/Sn)–O framework. The electrical properties were elucidated with emphasis on the ionic conduction mechanism in NdCaFeSnO_6_with charge carrier hopping at intermediate temperatures with an activation energy of 0.97 eV for grain boundaries and 0.31 eV for grains, based on impedance spectroscopy measurements which permit also to establish a–c and d–c conductivity contributions in this compound through Jonscher analysis. Furthermore, direct current *I*–*V* measurements revealed a Poole–Frenkel conduction mechanism at high temperatures with an activation energy of 1.55 eV. A comparison of these conduction processes was conducted to place this compound in the family of best materials for optoelectronic applications.

## Abstract 2

Crystal structure of NdCaFeSnO_6_.

## Graphical abstract

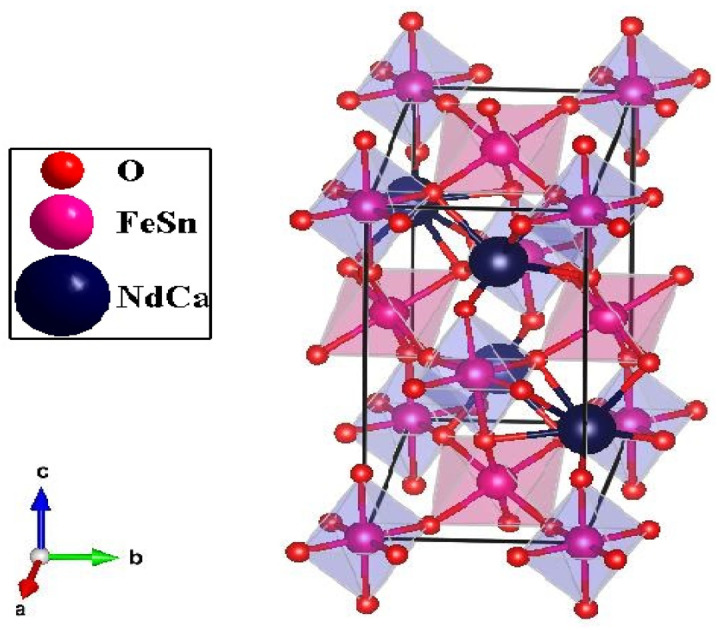

## 1. Introduction

The exploration and development of novel materials such as ceramics, nanorods, and composites exhibiting desirable physicochemical properties has remained a fundamental objective within the fields of chemistry and physics. These functional materials demonstrate significant potential for integration into various advanced technological systems, notably in energy storage, optoelectronic devices, photocatalysis, and supercapacitor technologies.^1–5^ Among them, materials adopting the perovskite crystal structure have been extensively investigated due to their exceptional structural tunability and multifunctionality. Perovskite-type compounds have demonstrated broad applicability in fields ranging from dielectric capacitors and solid oxide fuel cell anodes to heterogeneous catalysis and photovoltaic systems. The chemical flexibility of the perovskite structure allows for the formation of numerous elemental combinations at the A and B sites, resulting in simple (ABO_3_) or complex compositions (A′A″B_2_O_6_, A_2_B′B″O_6_, A′A″B′B″O_6_, *etc.*), depending on the atomic arrangement within the structure.

Among perovskite materials, calcium stannate (CaSnO_3_) has garnered significant scientific interest due to its noteworthy dielectric behavior and remarkable optical and electrochemical properties.^6^ One of its most remarkable attributes is its high upconversion efficiency, attributed to its low phonon energy, making it an optimal material for bright white upconversion luminescence. This enables the generation of a broad visible emission spectrum with minimal energy consumption under near-infrared excitation.^7–9^ Furthermore, CaSnO_3_ possesses a wide bandgap and a high melting point, making it suitable for various applications, including display matrices, thermally stable capacitors in electronics, ceramic materials, lithium-ion batteries, photocatalysis, and gas sensing.^8–10^ Sumithra *et al.* demonstrated that Fe doping in CaSnO_3_ significantly altered the material's structural, electronic, and magnetic properties.^11^ The incorporation of Fe ions induced lattice distortions, leading to modifications in bond lengths and lattice parameters. These structural changes facilitated the generation of localized defect states and enhanced magnetic interactions. The resulting alterations in the electronic structure also influenced the material's conductivity and optical characteristics, thereby affecting its overall magnetization and magnetic behavior. In particular, Ca(Fe_1−*x*_Sn_*x*_)O_3_ (ref. 11) has emerged as a promising candidate for spintronic and optoelectronic applications. Moreover, the incorporation of neodymium into the CaSnO_3_ structure induces structural modifications and charge compensation effects, enhancing the material's physical properties, making it suitable for high-level nuclear waste containment.^12^

Similarly, strontium stannate (SrSnO_3_) has attracted considerable attention due to its potential applications in single quantum flux circuits, lithium batteries, high-temperature humidity sensors, and low thermal capacitance capacitor devices.^13,14^ As an ideal transparent conducting oxide (TCO),^15^ SrSnO_3_ has been the subject of extensive research, focusing on doping and substitution strategies to improve its electro-optical properties.^16^ Recent studies in our laboratory have demonstrated that Al substitution at the B-site significantly enhances SrSnO_3_'s performance as a p-type TCO material.^17^ Moreover, it was reported that crystalline structure, electrical conductivity, optical, and dielectric properties of Sr_2_Sn_1.6_In_0.2_Cu_0.2_O_6−*δ*_ revealed its suitability for UV filtering, UV detection, and mixed ionic conduction in electronic applications.^18^ Given the industrial demand for innovative materials, B. Belgacem *et al.*^19^ identified Sr_2_(Sn_0.33_Sb_0.33_In_0.33_)_2_O_6_as a promising candidate for high-temperature optoelectronic and thermistor applications. Furthermore, the recently developed semiconductor nanomaterial La_0.25_Sr_0.75_Sn_0.4_In_0.25_Ru_0.35_O_3_, which features a narrow bandgap (1.3 eV) along with remarkable electrical and ferromagnetic properties, has demonstrated potential for applications in optoelectronics, memory devices, photocatalysis,^20^ and spintronics.^21^

In addition, double perovskites of the Sr_2_B′B″O_6_type, where B′ and B″ represent elements such as Ti, Co, and Mo, exhibit an intriguing p–n switching behavior as a function of temperature. This phenomenon is accompanied by significant variations in thermopower, making these materials suitable for the development of multifunctional devices. Due to their high electrical conductivity across a broad temperature range (300–1300 K), environmental compatibility, thermal stability, oxidation resistance, and lower processing costs compared to conventional chalcogenides and intermetallics, these compounds have been widely explored for thermoelectric applications.^22–24^

Furthermore, the La_2_CaFe_2_SnO_9_ double perovskite adopts a monoclinic structure (*P*2_1_/*n* space group) with an unusual cation ordering at the B-site. This material exhibits magnetic behavior characteristic of an iron-based magnet, with a Curie temperature exceeding room temperature. Notably, the Fe^3+^ magnetic moment decreases gradually upon heating from 5 K to room temperature.^25^

Building upon these findings, we propose the synthesis of NdCaFeSnO_6_(NCFSO) to harness the unique optical properties of rare-earth elements (Nd) while benefiting from the ferromagnetic nature of Fe^3+^ in its high-spin configuration (*t*_2g_^3^e_g_^2^). This study aims to explore the structural, optical, and electrical properties of this material, broadening its potential applications in energy storage technology and optoelectronic devices.

## 2. Experimental procedure

### 2.1. Synthesis protocol

The NdCaFeSnO_6_ sample was synthesized using the conventional solid-state reaction method, which involves high-temperature processing of precursor powders to obtain the desired crystalline phase. The starting materials, CaCO_3_, Nd_2_O_3_, Fe_2_O_3_, and SnO_2_ (purity > 99.95%), were carefully selected to ensure high chemical purity. Prior to synthesis, Nd_2_O_3_ was preheated at 1100 °C for 12 hours to eliminate any residual hydrogen carbonate (HCO_3_^−^) and moisture. The precursors were then weighed according to the stoichiometric ratio, thoroughly mixed, and ground in an agate mortar for 60 minutes to achieve a fine and homogeneous powder. The resulting powder was placed in an alumina crucible and calcined at 900 °C for 12 hours in a muffle furnace to initiate solid-state diffusion and phase formation. After cooling to room temperature, the powder was finely ground again and pressed into cylindrical pellets (13 mm in diameter, 2 mm in thickness) under an axial pressure of 10 t cm^−2^, ensuring proper grain boundary contact for enhanced densification. The compacted pellets were then placed in an alumina crucible and subjected to a sintering process at 1050 °C for 12 hours in ambient air to ensure adequate compactness and phase formation. Following this, the furnace was gradually cooled to 800 °C and held for 30 minutes before allowing the sample to return to room temperature. This controlled cooling step helped to minimize internal stresses and structural defects. Initial sintering resulted in a marked decrease in pellet diameter and thickness, confirming effective densification. To further optimize compaction and promote grain growth, a sequence of grinding, granulation, and additional sintering cycles was employed. Four successive sintering cycles at 1050 °C were conducted, with densification progress meticulously tracked *via* precise measurements of pellet diameter and thickness before and after each cycle. The convergence and subsequent stabilization of these parameters following the fourth cycle evidenced the achievement of a uniform, dense, and stable microstructure.

**Fig. 1:**
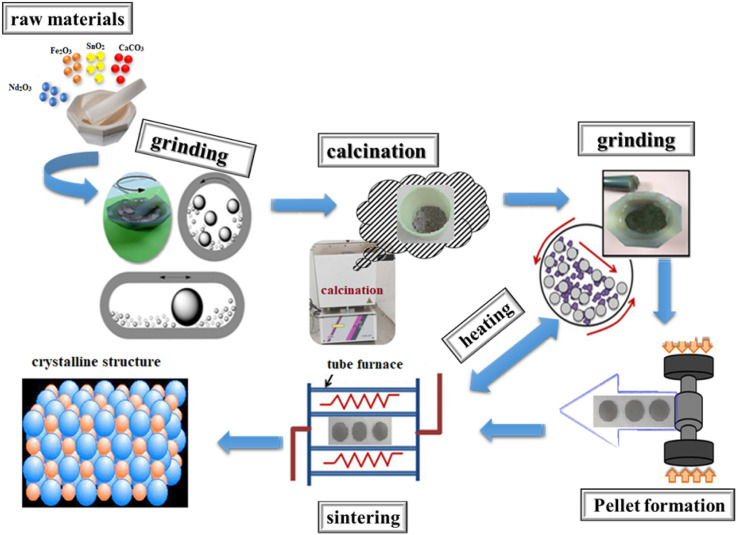
Schematical description of the elaboration process of NdCaFeSnO_6_ ceramics *via* the solid-state method.

Fig. 1 presents the schematic diagram of the solid-state synthesis process for the NdCaFeSnO_6_ sample fabrication. The stoichiometric proportions of the reagents used in this synthesis are represented by the following equation:1/2 Nd_2_O_3_ + CaCO_3_ + 1/2 Fe_2_O_3_ + SnO_2_ → NdCaFeSnO_6_ + CO_2(g)_

After each heat treatment, the sample undergoes powder X-ray diffraction (XRD) analysis to verify the formation of the desired phase and confirm its purity.

### 2.2. Characterization techniques

X-ray diffractograms were obtained using an X'Pert^3^ Powder PANalytical diffractometer in Bragg–Brentano geometry, employing monochromatic Kα radiation with a copper anticathode (*λKα*_1_ = 1.54056 Å). The scanning was performed in the 20° ≤ 2*θ* ≤ 110° range, with a step size of 0.01° and a counting time of 30 seconds per step. The diffraction pattern refinements were carried out using the FULLPROF program.^26^

The morphological analysis of the synthesized sample was carried out using a field emission scanning electron microscope (FE-SEM, FEI Quanta FEG 200) equipped with an energy-dispersive X-ray spectroscopy (EDX) system. SEM observations were performed at an accelerating voltage of 10 kV, providing adequate resolution for detailed examination of the sample's microstructural characteristics. Micrographs were registered at various magnifications ranging from 9195× to 150 00×, enabling the assessment of grain morphology, distribution, and surface texture.

Raman vibration modes were identified using a RENISHAW in *Via* Raman spectroscope (Renishaw UK Sales Ltd, Gloucestershire, UK) with a non-polarized laser (*λ* = 633 nm). To minimize sample heating and prevent any thermal damage at the surface, a 0.3 neutral density (ND) filter was used, reducing the effective laser power to approximately 5% of its nominal intensity. For each measurement range, five consecutive accumulations were performed, with an exposure time of 30 seconds per accumulation, in order to enhance the signal-to-noise ratio. This procedure was repeated in three independent acquisitions obtain complete acquisition.

The current–voltage (*I*–*V*) characteristics were obtained using a KEITHLEY SourceMeter 2611A System (Tektronix/Keithley, Cleveland, OH, USA), with the sample connected to a Linkam THS340-type oven (Linkam Scientific Instruments Ltd, Redhill, UK).

UV-visible diffuse reflectance spectra (DRS) were acquired using a PerkinElmer Lambda 365 spectrophotometer, covering the 200–1100 nm range. By analyzing the DR spectra, key optical properties such as band gap energy and light absorption behavior were determined, contributing to a comprehensive characterization of the material.

Finally, *I*–*V* measurements were performed on the NdCaFeSnO_6_ sample at a specified temperature to investigate the conduction mechanisms involved, using Keithley 2611 SMU Multimeter.

For this purpose, commercial silver electrodes were deposited on both surfaces of the sintered ceramic pellets. The coated samples were air-dried for 30 minutes and then heat-treated at 300 °C for 3 hours to ensure good adhesion and establish stable ohmic contact.

## 3. Results and discussions

In this section, we will explore the results of our characterization study, beginning with an analysis of the structural properties and concluding with an investigation of the conduction mechanism.

### 3.1. X-ray diffraction study

#### 3.1.1. Structural refinement of NdCaFeSnO_6_

Recent studies conducted in our laboratory have focused on the double perovskite stannate oxide (La_2/3_Sr_1/3_)_2_(Sn_1/3_Fe_1/3_Cu_1/3_)_2_O_6−*δ*_.^27^ As part of this research, we are currently exploring neodymium, a rare earth element known for its excellent luminescent properties. In order to introduce the desired functionality of Nd and Fe elements into the CaSnO_3_ parent compound, we synthesized the NdCaFeSnO_6_ sample after incorporating rare earth elements in the A-site (r_Nd_^3+^≈ r_Ca_^2+^ ≈ 1.3 Å) and transition metals in the B-site (r_Fe_^3+^ ≈ r_Sn_^4+^ ≈ 0.65 Å).

Several searches conducted on the COD, PDF4+, and highscore databases confirmed the novelty of our synthesized compound. Furthermore, these searches enabled us to determine the most suitable structural model, which is prototyped from the double perovskite compound Ca_2_CoNbO_6_,^28^ exhibiting monoclinic symmetry with space group *P*2_1_/*n*.

To further identify and confirm the structure of the NdCaFeSnO_6_ compound, a slow scan was performed to collect a highly resolved XRD pattern. The most intense peaks were indexed according to monoclinic symmetry (*P*2_1_/*n*), although this group is not standard. Therefore, we opted for the *P*2_1_/*c* space group, and the reliability factors confirmed this choice.

An important parameter in the ABO_3_ perovskite-like structure is the Goldschmidt tolerance factor (*t*), which is related to the likelihood of forming octahedral coordination of the B and B′ cations with the oxygen anion, as well as the tilting of these octahedra. Based on the effective Shannon ionic radii,^29^*r*_Nd^3+^_ = 1.27 Å, *r*_Ca^2+^_ = 1.34 Å (coordination 12), *r*_Sn^4+^_ = 0.69 Å, *r*_Fe^3+^_ = 0.645 Å (high spin), and *r*(O^2−^) = 1.40 Å, the to lerance factor for NdCaFeSnO_6_ structural organization was determined based on this formula:
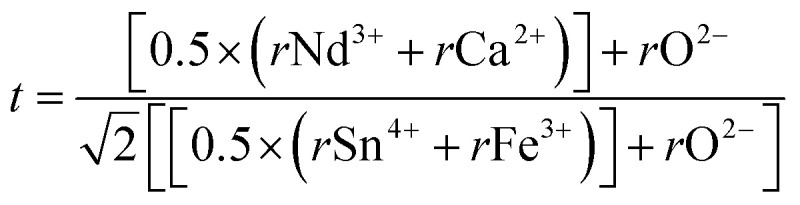


The calculated tolerance factor value found equal to 0.925 falls within the stability range of 0.825 to 1.06,^30^ confirming that this sample exhibit a perovskite-like structural stability. However, the deviation from unity suggests significant distortions from the ideal cubic perovskite structure. These distortions arise due to variations in the ionic radii at sites A and B, leading to minor octahedral tilting and a reduction in symmetry.

**Fig. 2:**
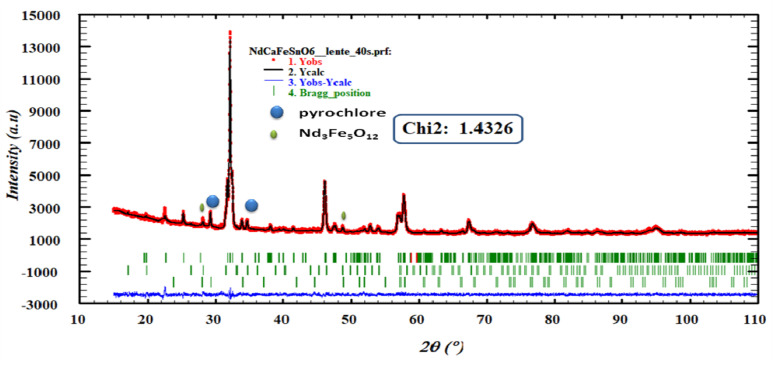
Rietveld refinement pattern of NdCaFeSnO_6_ (the black line corresponds to the calculated pattern, red solid dotted corresponds to experiment and the blue solid line is the difference between the experimental and the fitted values). The green tick marks correspond to the expected Bragg positions: the top tick marks correspond to the main NdCaFeSnO_6_ phase, the middle tick marks to the pyrochlore (Nd_2_Sn_2_O_7_) phase, and the bottom tick marks to the Nd_3_Fe_5_O_12_ phase.

Additionally, the octahedral factor µ was determined using the equation:
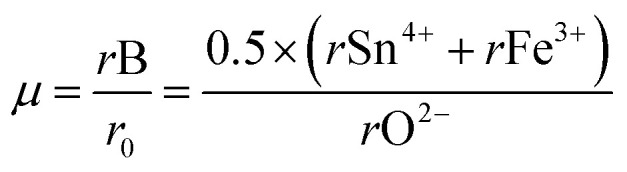


The obtained value of 0.4767 lies within the stability range (0.414–0.732),^31^ indicating the formation of stable (Sn/Fe)O_6_ octahedra. This result, in conjunction with the tolerance factor, supports the prediction that NdCaFeSnO_6_ adopts a stable double perovskite structure. The structural refinement of the NdCaFeSnO_6_ sample, performed using the FullProf software with the Rietveld method, confirms that the compound crystallizes in the monoclinic system (space group *P*2_1_/*c*). The analysis indicates a predominant phase with a 1:1 ratio of B-site cations. Additionally, a minor secondary phase (5.33%) is identified, which is likely attributed to pyrochlore Nd_2_Sn_2_O_7_. A negligible fraction (0.8%) of Nd_3_Fe_5_O_12_ is also detected. The observed, calculated and difference profiles for the Rietveld refinement of NdCaFeSnO_6_ compound was shown in Fig. 2. The lattice parameters refined by the Rietveld method are listed in Table 1.

Rietveld refinement results for NdCaFeSnO_6_ at room temperature

**Table:** 

*R*_B_ = 3.52%	*a* = 5.503(1) Å	Alpha = 90°
*R*_F_ = 4.03	*b* = 5.627(5) Å	Beta = 125.045(1)°
*χ* ^2^ = 1.43	*c* = 9.591(5) Å	Gamma = 90°
Fract (%): 94.67	*V* (Å^3^) = 243.072(1)	

a *B*_iso_: isotropic atomic displacement parameter.

b Occ.: site occupancy factor.

**Table:** 

Atomes	*X*	*Y*	*Z*	*B*_iso_ (Å^2^)^a^	Occ^b^	Site
O1	0.153(9)	0.017(2)	0.251(8)	1.517(4)	1.0	4e
O2	0.208(6)	0.746(5)	0.030(4)	0.068(9)	1.0	4e
O3	0.310(7)	0.214(4)	0.065(4)	0.556 (8)	1.0	4e
Fe1	1/2	0	1/2	0.879(0)	0.5	2d
Sn1	1/2	0	1/2	0.879(0)	0.5	2d
Fe2	0	0	0	0.565(0)	0.5	2a
Sn2	0	0	0	0.565(0)	0.5	2a
Nd	0.265(2)	0.4519(3)	0.2522(2)	0.643(7)	0.5	4e
Ca	0.265(2)	0.4519(3)	0.2522(2)	0.643(7)	0.5	4e

The double perovskite structure of NdCaFeSnO_6_ refers to a complex oxide material characterized by two interwoven perovskite-like substructures. Based on the Ca_2_CoNbO_6_ isotype,^28^ the structural model of NdCaFeSnO_6_ presents an ordered distribution of cations at specific lattice sites:

• The 4e site accommodates the larger cations Nd^3+^ and Ca^2+^, which are statistically equivalent at the A-site positions. These sites are spacious enough to accommodate large ions.

• The B-site positions (2a (0,0,0) and 2d (1/2,0,1/2)) are occupied by the Fe^3+^ and Sn^4+^ cations, which form corner-sharing octahedral coordination with surrounding oxygen atoms.

• The oxygen ions (O^2−^) fill the three 4e sites, coordinating with metal cations and enhancing the overall stability of the perovskite lattice.

**Fig. 3:**
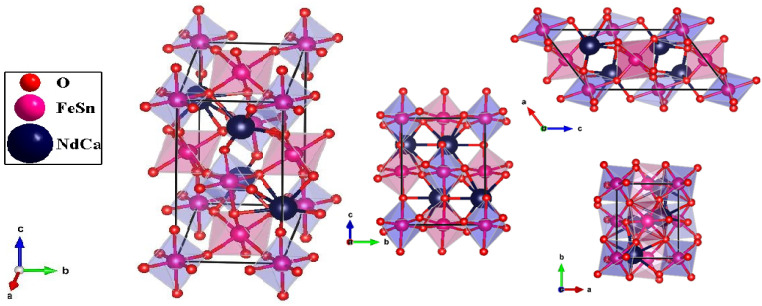
Crystal structure of NdCaFeSnO_6_ at different lattice orientation.

**Fig. 4:**
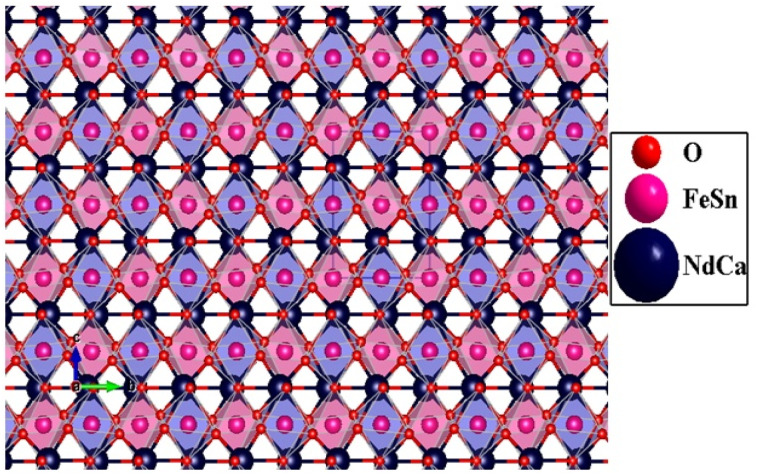
A perspective view of the NdCaFeSnO_6_ structure.

The NdCaFeSnO_6_ structure presented in Fig. 3 consists of a complex arrangement of rare-earth, alkaline-earth, transition metal, and oxygen ions in a perovskite-related framework. It can be visualized as an alternating stack of two layers:

1. Octahedral layer – composed of rows of (Sn_1_/Fe_1_)O_6_ octahedra (light blue) and (Sn_2_/Fe_2_)O_6_ octahedra (pink), aligned along the *b*-axis.

2. Rock-salt-type oxide layer – formed by alternating Nd/CaO units along the same axis (see Fig. 4).

This unique arrangement contributes to a range of intriguing physical properties, particularly in terms of conductivity and magnetism, making NdCaFeSnO_6_ a promising material for various applications. Interatomic distances and angles for polyhedron are listed in Table 2.

**Table 2:** Interatomic distances and angles for polyhedron in NdCaFeSnO_6_ structure

Monoclinic *P*2_1_/*c*	Distance (Å)	Angles (°)
2 × (Sn1/Fe1)–O1	1.84(3)	1 × O1– (Sn1/Fe1)–O1 180(3)
2 × (Sn1/Fe1)–O2	2.27(6)	2 × O1–(Sn1/Fe1)–O2 87(2)
2 × (Sn1/Fe1)–O3	1.948(1)	2 × O1–(Sn1/Fe1)–O2 93(4)
		2 × O1–(Sn1/Fe1)–O3 88.6(1)
		2 × O1–(Sn1/Fe1)–O3 91.4(1)
		1 × O2–(Sn1/Fe1)–O2 180(5)
		2 × O2–(Sn1/Fe1)–O3 98(2)
		2 × O2–(Sn1/Fe1)–O3 82(3)
		1× O3–(Sn1/Fe1)–O3 180.0(1)
Moyen <(Sn1/Fe1)–O>	2.019(3)	
2 × (Sn2/Fe2)–O1	2.22(4)	1 × O1–(Sn2/Fe2)–O1 180(3)
2 × (Sn2/Fe2)–O3	1.78(4)	2 × O1–(Sn2/Fe2)–O2 86(4)
	2.151(1)	2 × O1–(Sn2/Fe2)–O2 94(3)
		2 × O1–(Sn2/Fe2)–O3 87(2)
		2 × O1–(Sn2/Fe2)–O3 93.0(1)
		1 × O2–(Sn2/Fe2)–O2 180(4)
		2 × O2–(Sn2/Fe2)–O3 79(2)
		2 × O2–(Sn2/Fe2)–O3 101(3)
		1 × O3–(Sn2/Fe2)–O3 180.0(1)
Moyen <(Sn2/Fe2)–O>	2.050(1)	
(Ca/Nd)–O1	2.492(2)	
(Ca/Nd)–O1	3.259(1)	
(Ca/Nd)–O1	2.36(4)	
(Ca/Nd)–O1	3.22(4)	
(Ca/Nd)–O2	2.65(7)	
(Ca/Nd)–O2	2.29(4)	
(Ca/Nd)–O2	3.07(4)	
(Ca/Nd)–O2	3.28(8)	
(Ca/Nd)–O3	2.61(3)	
(Ca/Nd)–O3	2.326(1)	
(Ca/Nd)–O3	3.550(1)	
(Ca/Nd)–O3	2.79(2)	
Moyen <(Ca/Nd)–O>	2.824(7)	

#### 3.1.2. Crystallite size determination

The crystallite sizes of NdCaFeSnO_6_ powder were determined using the Scherrer equation:1
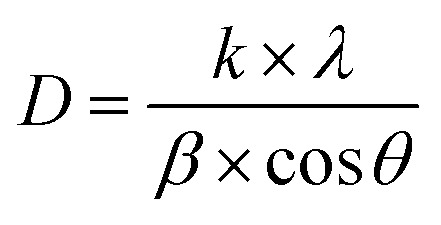
where: *D* represents the crystallite size (in nm), *k* is a shape factor constant (0.94 for spherical crystals), *λ* is the wavelength of X-ray radiation (Cu-Kα = 1.54056 Å), *β* is the full width at half maximum (FWHM) of the most prominent diffraction peaks (in radians), *θ* is the Bragg diffraction angle for the corresponding (*hkl*) plane.

The most significant diffraction peaks (as listed in Table 3) were used for this calculation. The average crystallite size was determined to be 34.106(4) nm, closely matching that of CaSnO_3_ doped with 1% Fe, which crystallizes in an orthorhombic structure (space group *Pnma*).^11^

**Table 3:** The most intense peaks, with their corresponding (*hkl*) and FWHM for NdCaFeSnO_6_ compound

*h*	*k*	*l*	2*θ*	*I*_obs_	FWHM
0	0	2	22.6395	5.8	0.17773
1	1	0	25.3098	61.7	0.17898
0	2	0	31.7765	509.2	0.18430
1	1	1	32.2047	1085.7	0.18477
0	2	1	33.8144	81.6	0.18665
−2	0	2	32.5142	522.8	0.18511
−2	1	1	38.1511	20.4	0.19274
−2	2	2	46.1028	572.6	0.20770
0	0	4	46.2289	309.6	0.20798
−2	2	1	47.6261	38.6	0.21113
2	2	0	51.9727	15.9	0.22188
−1	1	5	52.0193	3.4	0.22201
1	3	0	52.8268	55.5	0.22417
−3	1	1	57.8328	242.8	0.23870
2	2	2	67.3812	151.1	0.27196
1	1	5	76.7363	73.3	0.31217
4	0	0	86.3469	30.1	0.36263
4	2	0	94.9437	30.5	0.41748

Although the co-substitution of elements with different ionic radii inevitably induces micro-deformations within the lattice, these distortions significantly influence the average crystallite size. To account for both crystallite size effects and lattice strain, Williamson and Hall proposed a method that separates these two contributions to peak broadening in nanocrystals. This approach can be mathematically expressed as:2
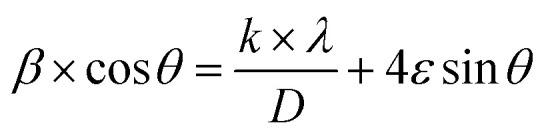
where: *β* is the full width at half maximum (FWHM) of the diffraction peak (in radians), *θ* is the Bragg diffraction angle, *k* is the shape factor constant (typically 0.94), *λ* is the X-ray wavelength (Cu-Kα = 1.54056 Å), *D* is the average crystallite size (in nm), *ε* is the lattice strain.

By plotting *β* cos(*θ*) against 4 sin(*θ*), the slope of the resulting linear fit provides *ε* (lattice strain), while the *y*-intercept yields the inverse of the crystallite size (*D*^−1^). This method allows for a more precise assessment of both structural distortions and size-dependent broadening effects in NdCaFeSnO_6_.

**Fig. 5:**
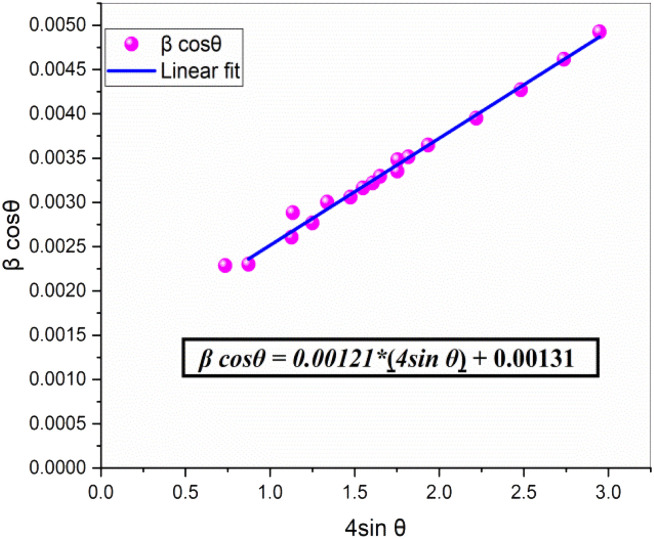
Williamson–Hall plot for estimating the crystallite size and lattice strain of the NdCaFeSnO_6_ compound. The slope represents the lattice strain (*ε*), while the *y*-intercept provides an estimate of the crystallite size (*D*).

This model assumes a homogeneous lattice strain in all directions, with *ε* representing the intrinsic strain. To analyze this, a graph was plotted with β cos(*θ*) on the *y*-axis and 4 sin*θ* on the *x*-axis, incorporating all Bragg peaks. The slope of the fitted line represents the overall lattice strain (*ε*), while the *y*-intercept provides an estimate of the crystallite size (*D*). The resulting fit is illustrated in Fig. 5.

The calculated micro-strain value for the NdCaFeSnO_6_ compound is 1.21 × 10^−3^, which is lower than that observed for the parent CaSnO_3_ and Fe-doped compounds (Fe-1%, Fe-3%, and Fe-5%), recorded at 1.812 × 10^−3^, 1.631 × 10^−3^, 1.522 × 10^−3^, and 1.413 × 10^−3^, respectively.^11^ This reduction can be attributed to the differences in ionic radii of the constituent elements in NdCaFeSnO_6_: *r*(Nd^3+^) = 1.27 Å, *r*(Ca^2+^) = 1.34 Å (site A), *r*(Sn^4+^) = 0.69 Å, and *r*(Fe^3+^) = 0.645 Å (site B).

The crystallite size determined using the Williamson–Hall method is 96.972(9) nm, which notably differs from the value obtained *via* the Scherrer equation. A comparative analysis, as highlighted in a study published in Advances in Natural Sciences: Nanoscience and Nanotechnology (2019),^32^ demonstrates that the Scherrer method neglects lattice deformations, often underestimating crystallite sizes, as seen in this case. In contrast, the Williamson–Hall approach, which decouples the effects of crystallite size and lattice strain, provides a more accurate estimation, particularly in materials with significant internal stresses.

The substantial discrepancy between the two methods underscores the presence of notable internal strains within the NdCaFeSnO_6_ double perovskite compound. In summary the crystallite size and micro-strain values determined *via* Williamson–Hall analysis confirm the nanometric scale of the NdCaFeSnO_6_ powder. The observed micro-strain, evidenced by XRD peak broadening and corroborated by microstructural features, significantly influences the material's optoelectronic and dielectric behavior. These localized lattice distortions alter orbital interactions, particularly within the (Sn/Fe)O_6_ octahedra, leading to bond angle deformations and promoting the generation of point defects such as oxygen vacancies. These defects directly affect charge carrier mobility, dielectric relaxation mechanisms, and contribute to the observed bandgap narrowing.

### 3.2. Scanning electron microscopy (SEM) and EDX analysis

**Fig. 6:**
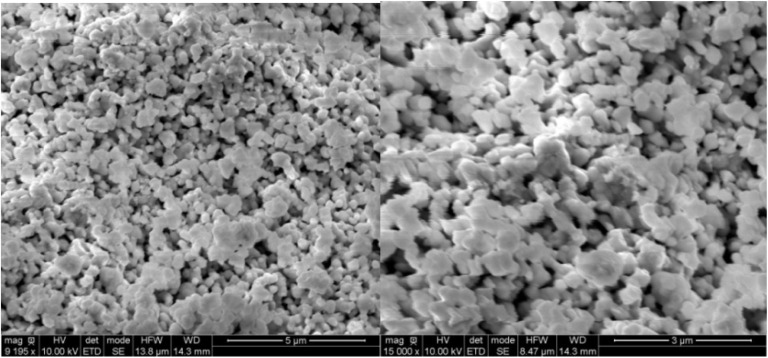
SEM micrographs at 9195 and 15 000 magnifications.

This qualitative and semi-quantitative study enabled the identification and verification of all elements present in the NdCaFeSnO_6_ double perovskite compound. Fig. 6 presents micrographs registered at magnifications 9195× and 150 00×, illustrating a homogeneous elemental distribution and a well-organized structural arrangement. The powder demonstrates a high level of densification with uniformly shaped grains indicating consistent morphology across the sample.

Additionally, the sample consists of particles of varying sizes, with most having a diameter of approximately 0.15 µm, while a few reached up to 0.4 µm. To determine the elemental composition of the synthesized powder, EDX analysis was performed. The results confirm the presence of all expected chemical elements in their stoichiometric proportions, further validating the successful synthesis of the NdCaFeSnO_6_ double perovskite compound.

**Fig. 7:**
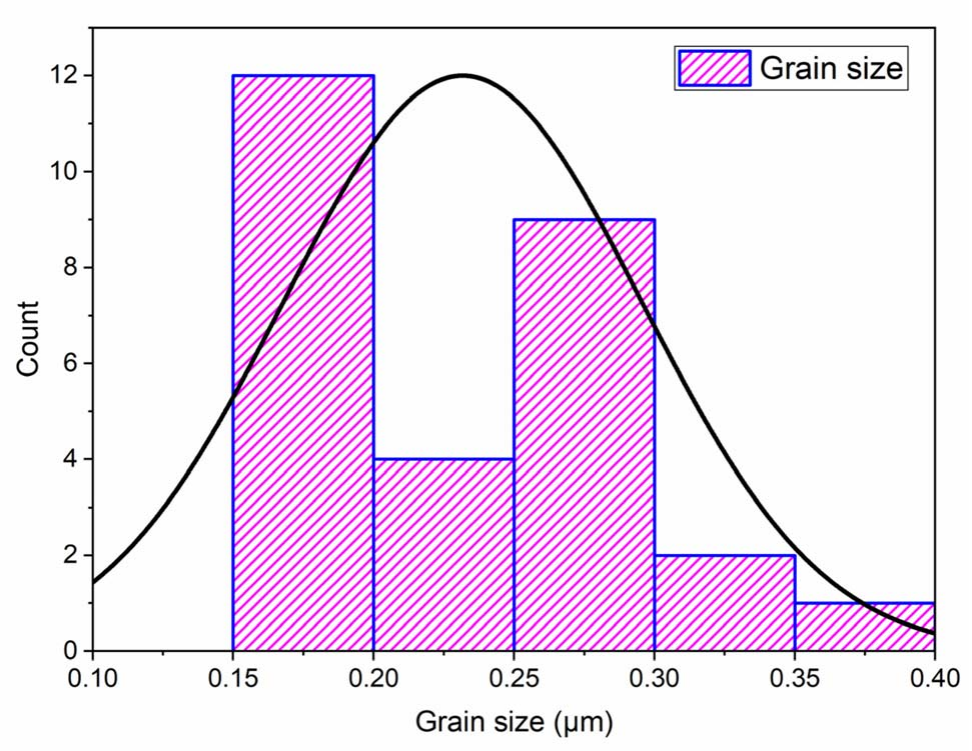
Histogram of the grain size distribution for NdCaFeSnO_6_.

Grain size distribution was quantitatively assessed using Image J software.^33^ Measurements were conducted on a representative set of at least 28 grains. As shown in the histogram (Fig. 7), the average particle size of the NdCaFeSnO_6_ powder is approximately 226 nm, which is significantly larger than the values obtained from XRD analysis. This discrepancy arises from the differences in measurement techniques: scanning electron microscopy (SEM) provides an estimation of particle size, often reflecting the size of agglomerates, whereas X-ray diffraction (XRD) determines the crystallite size, representing the individual grains within a particle.

**Fig. 8:**
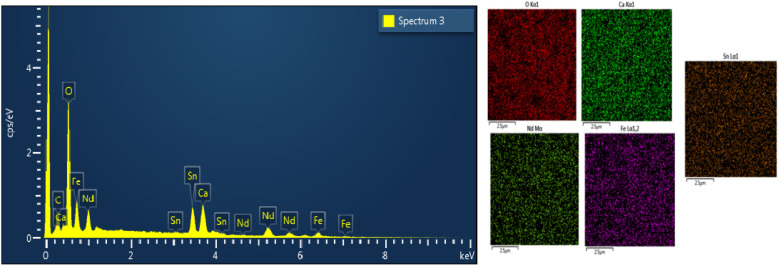
EDX spectrum of NdCaFeSnO_6_ compound.

Fig. 8 presents the EDX spectrum for the NdCaFeSnO_6_ sample. The EDX analysis, which is qualitative and semi-quantitative in nature, enabled the identification of all the constituent elements of the NdCaFeSnO_6_ compound. However, since this technique is primarily surface-sensitive and has a shallow analysis depth, the results mainly reflect the surface composition. Within these limits, the data confirm the expected stoichiometry. Moreover, any potential interfering phase detected corresponds to a combination of Nd, Ca, Fe, Sn, and O elements.

From EDX (Energy Dispersive X-ray Spectroscopy) analysis, we use separate individual color to distinguish different elements present in a sample. The software assigns specific colors to different elements based on their characteristic X-ray emissions. This visualization technique is crucial for identifying the spatial distribution of elements within a material. Fig. 8 shows the presence of the element present in the studied compound.

This verification confirms the successful synthesis of the NdCaFeSnO_6_ double perovskite compound with the expected stoichiometry. The EDX analysis conducted at four different locations ensures that the elemental distribution is homogeneous across the sample. Any minor deviations from the theoretical composition could be attributed to instrumental limitations or localized variations in elemental concentration. To evaluate the synthesis quality, the percentage deviations from the theoretical stoichiometric values were calculated, as shown in Table 4. The low absolute errors confirm the compositional accuracy of the synthesized NdCaFeSnO_6_ compound.

**Table 4:** Mass percentages of chemical elements obtained by EDX analysis

Element	Mass percentage (wt%)	Experimental composition	Theoretical composition	Absolute error (%)
O	21.34	6.29	6.00	0.05
Ca	8.5	1	1.00	0
Nd	32.16	1.05	1.00	0.05
Fe	12	1.01	1.00	0.01
Sn	26	1.03	1.00	0.03

### 3.3. Raman spectroscopy

Raman spectroscopy is a powerful, non-destructive technique widely used to investigate various material properties, including crystalline quality, the structure of inorganic compounds, and the presence of disorder and defects in semiconductor oxides. This technique relies on the inelastic scattering of light, known as the Raman effect. The number of bands observed in a Raman spectrum depends on the crystal symmetry of the studied compound and corresponds to the vibrational modes of the atoms within the structure.

In this study, the crystal symmetry and local distortion of the synthesized compound NdCaFeSnO_6_ were examined through Raman spectroscopy at room temperature. Spectra were recorded in the range of 50 to 900 cm^−1^ using an excitation wavelength of 633 nm. To accurately distinguish between vibrational and translational lattice modes, a polarized Raman spectrum was required.^34^ For a monoclinic structure with space group *P*2_1_/*c* (*C*^5^_2h_), the Raman-active modes are given by: Γ (*P*2_1_/*c*) = 6T (3Ag + 3Bg) + 6L (3Ag + 3Bg) + 2ν_1_ (Ag + Bg) + 4ν_2_(2Ag + 2Bg) + 6ν_5_ (3Ag + 3Bg).^32^

The translational lattice modes (T) associated with non-degenerate bands exhibit minimal Raman activity in the low-frequency range of 50–200 cm^−1^. The vibrational lattice modes (L), also referred to as silent modes, were observed in the 200–400 cm^−1^ range. Meanwhile, the internal modes ν_1_, ν_2_, and ν_5_ displayed relatively high Raman activity above 400 cm^−1^.

**Fig. 9:**
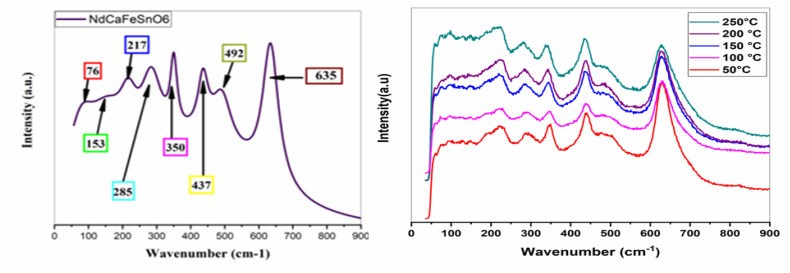
Room temperature Raman spectra of NdCaFeSnO_6_ sample.

The co-substitution of Nd and Fe in the CaSnO_3_ compound may induce shifts in the Raman peak frequencies, particularly in the low-to intermediate-frequency ranges, due to changes in crystal symmetry. However, the overall vibrational characteristics and the Raman mode pattern are expected to remain consistent with those of other double perovskite-type compounds exhibiting *P*2_1_/*c* symmetry.^35–37^ The experimental Raman spectrum recorded at room temperature on NdCaFeSnO_6_ ceramic, presented in Fig. 9, provides precise frequency values for the observed vibrational modes. This spectrum measured in the range 50–900 cm^−1^ using a 633 nm excitation wavelength, reveals several characteristic vibrational modes of the *P*2_1_/*c* monoclinic structure. In the low-frequency region (50–200 cm^−1^), the translational modes (T) associated with Nd and Ca cations at the A-site exhibit weak Raman activity. Two distinct bands are observed at 76 cm^−1^ and 153 cm^−1^, corresponding to the translation of these cations and the slight tilting of the (Sn/Fe)O_6_ octahedra, respectively. The vibrational modes (L), also known as silent modes, appear in the 200–400 cm^−1^ range. Although these modes are typically Raman-inactive, they become detectable due to the symmetry reduction induced by the monoclinic structure. Three main bands are identified in thisregion:

• 217 cm^−1^: attributed to bending vibrations involving oxygen displacement.^38^

• 285 cm^−1^: associated with Nd–O scattering, in agreement with previous studies.^12^

• 350 cm^−1^: linked to the distortion of the octahedral coordination around Sn and Fe cations.^11^

In the high-frequency region (>400 cm^−1^), the internal modes ν_1_, ν_2_, and ν_5_ exhibit strong Raman activity:

• 437 cm^−1^: assigned to the ν_5_ mode, corresponding to the bending motion of O–(Sn/Fe)–O bonds.

• 492 cm^−1^ and 635 cm^−1^: attributed to the ν_2_ and ν_1_ modes, corresponding to the symmetric and asymmetric stretching vibrations of the (Sn/Fe)O_6_ octahedra, respectively. These results are consistent with previous Raman studies.^39–41^

Fig. 9 shows the Raman spectra at different temperature. We note an expansion of the crystal lattice as a function of temperature resulting in the progressive displacement of the modes, in particular the one located at 350 cm^−1^. No mode appears or disappears, proving that there is no clear phase transition in the material (no symmetry breaking).

Table 5 summarizes the experimentally observed vibrational frequencies, their possible assignments, and their correlation with the crystal structure and characteristic spectral signatures of the material.

**Table 5:** Raman mode related to NdCaFeSnO_6_ lattice dynamics in *P*2_1_/*c* symmetry group

Raman wavenumber (cm^−1^)	Modes	Attributes
76	T	•Translation of Nd and Ca cations from A-site
153		•Low tilt of (Sn/Fe)O_6_ octahedra
217	L	•Bending vibrations
285		•Nd–O scattering
350		•Distortion of the octahedral coordination around the Sn and Fe cations
437	ν_5_	•The internal oxygen bending motion of the O–(Sn/Fe)–O
492	ν_2_	•Totally symmetric and antisymmetric stretching of the (Sn/Fe)O_6_ octahedron
35	ν_1_	

### 3.4. UV-visible analysis

To investigate the optical properties of our compound, we performed UV-vis spectroscopy using a solid-state UV-visible spectrophotometer.

**Fig. 10:**
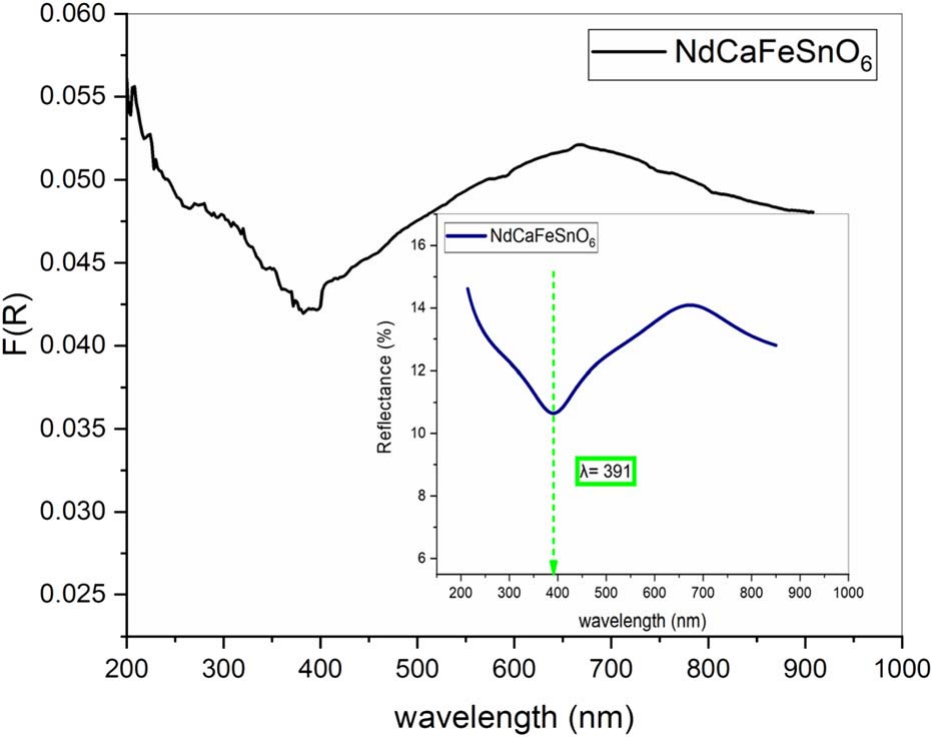
Diffuse reflectance spectrum and Kubelka–Munk function *F*(*R*) of NdCaFeSnO_6_.

The reflectance spectrum of the NdCaFeSnO_6_ sample, presented in Fig. 10, exhibits an optical response characteristic of a wide bandgap semiconductor, with strong absorption occurring in the 350–450 nm region, peaking at approximately 391 nm.

**Fig. 11:**
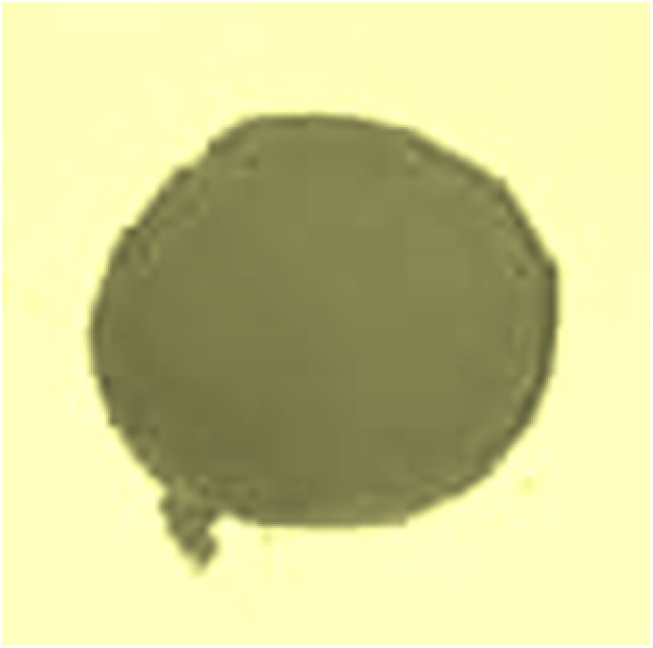
The actual color of the NdCaFeSnO_6_ compound pellet.

The sample demonstrates intense optical absorption in the 380–430 nm wavelength range, which corresponds to the violet region of the visible spectrum. As a result, the sample appears green, the complementary color of violet. Fig. 11 depicts the actual color of the NdCaFeSnO6 pellet, consistent with its absorption characteristics.

The reflectance spectra were further analyzed using the Kubelka–Munk theory to determine the maximum absorbance of the sample.^42^ The Kubelka–Munk function was calculated based on the measured reflectance values using the following equation:

3 *F*(*R*) = (1 − *R*)^2^/2*R* ≈ *K*/*S*

where *S* and *K* represent the scattering and absorption coefficients, respectively, while *R* denotes the diffuse reflectance.

The optical band gap (*E*_g_) of NdCaFeSnO_6_ was determined using the Tauc plot method. The Tauc equation is given by:

4 (*F*(*R*) × *hν*)^*p*^ = *A*(*hν* − *E*_g_)

where *A* is a constant, *hν* is the incident photon energy, *E*_g_ is the band gap, and *p* is an exponent that depends on the type of electronic transition. The value of *p* distinguishes the nature of the optical transition:

• *p* = 2 for an allowed direct transition.

• *p* = 1/2 for an allowed indirect transition.

**Fig. 12:**
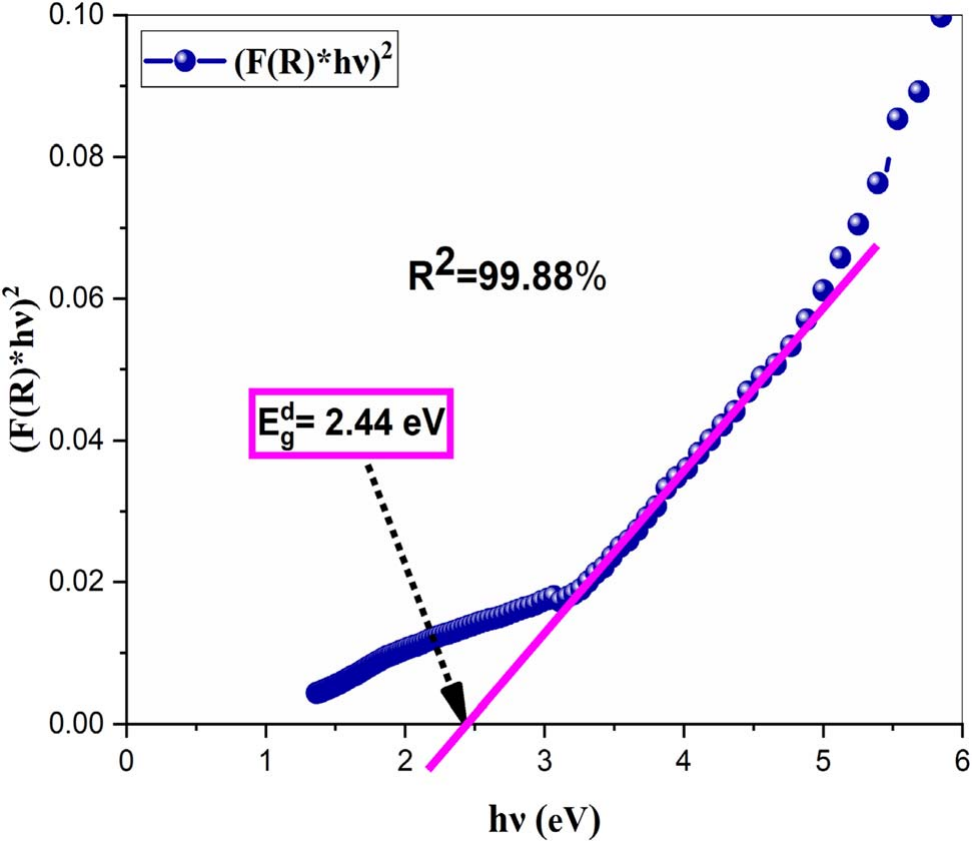
Tauc plot for direct bandgap for the NdCaFeSnO_6_.

In Fig. 12, we plot (*F*(*R*) *hν*)^2^ as a function of *hν* (incident photon energy). The band gap energy values are obtained from the intersection of the extrapolated linear region with the photon energy axis (*hν*).

Fig. 12 presents the Tauc plot corresponding to the direct electronic transitions since the obtained Tauc parameter is 2 for NdCaFeSnO_6_ nanoparticles. The corresponding gap energy was 2.44 eV. These values are relatively low compared to the parent compound CaSnO_3_,^43^ which exhibits an ultrawide band gap of 4.2–4.4 eV, as well as Fe-doped CaSnO_3_ compounds,^11^ which have band gap values ranging from 3.04 to 3.78 eV.

The optical band gap is primarily influenced by intermediate energy levels within the band gap, as well as the degree of order and disorder in the compound's structural composition. In this case, the band gap reduction (*E*_g_) can be attributed to the presence of oxygen vacancies 
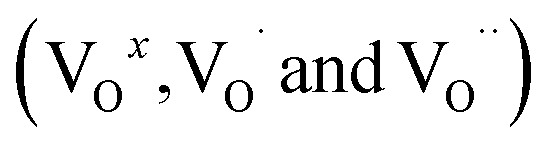
, lattice defects, and local bond distortions.^44^ As reported in the literature,^11^ substituting Sn with Fe leads to the formation of stable holes at the top of the valence band and increases the covalency of the Sn–O bond. Moreover, optical absorption studies confirm the incorporation of Fe into our structure.^45^

### 3.5. Dielectric properties, impedance spectroscopy and *I*–*V* measurements

#### 3.5.1. Dielectric properties

Dielectric spectroscopy measurements were performed on NdCaFeSnO_6_ ceramics. Pellets are compacted as a disk having thickness of 0.55 mm and a surface of 19.86 mm^2^. Silver paste was deposited at both the two faces, followed by three hours thermal annealing. A relative ceramic pellets density of 6.205 g cm^−3^ was calculated using the Archimede's method. This value almost is close to the theoretical value (6.215 g cm^−3^) corresponding to a relative density of approximately ∼99%. Such a high-level densification ensures excellent compact microstructure, which is crucial for reliable and reproducible electrical characterization.

**Fig. 13:**
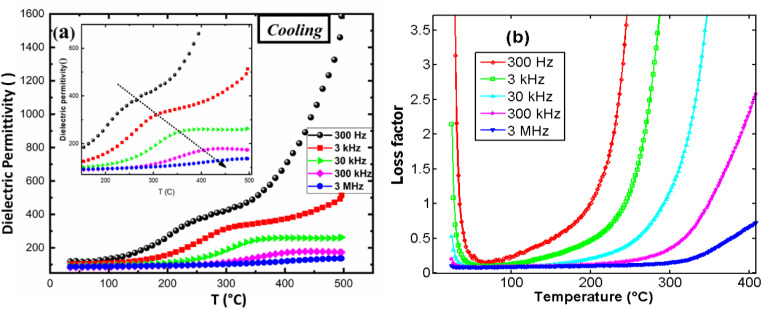
(a) Real dielectric permittivity, (b) dielectric loss, *versus* temperature for several frequencies.

Measurements were performed using wire connecting the samples to Solartron SI-1200 apparatus. Fig. 13(a) illustrates the behavior of the real part of the dielectric permittivity during the cooling process as a function of temperature, for various frequencies. The onset of a conductivity regime is observed to depend on the source frequency. At higher frequencies, the onset of this regime is delayed to higher temperatures. A noticeable bump appears in the permittivity curve, followed by a sharp increase in permittivity, when temperature increases, which is attributed to the electrical conductivity in the material. This behavior is further confirmed by the high dielectric loss observed in the material (Fig. 13(b)), which is significantly pronounced at lower frequencies and lower temperatures. This can be attributed to ionic conductivity and thermal charge carrier activation in the material. Dielectric permittivity was found around 120 at room temperature and 3 kHz.

**Fig. 14:**
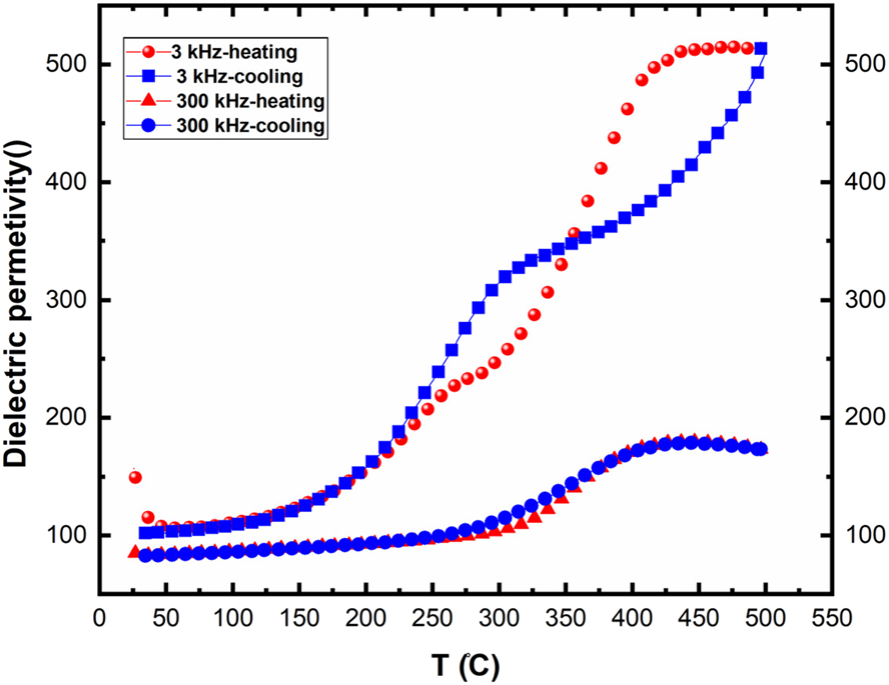
Thermal hysteresis observed of real permittivity at the frequencies 30 kHz and 300 kHz.

Fig. 14 presents the variation of real permittivity during heating and cooling at 3 kHz and 300 kHz. Two distinct dynamic behaviors are observed at these frequencies, with the presence of two thermal hysteresis loops at the lower frequency (30 kHz). At heating, two separated anomalies were observed for the frequency 3 kHz. However, only one anomaly is observed for the frequency 30 kHz. The second anomaly (at high temperature) disappeared at cooling. All these behaviors are in favor of electrical conductivity in this compound.

**Fig. 15:**
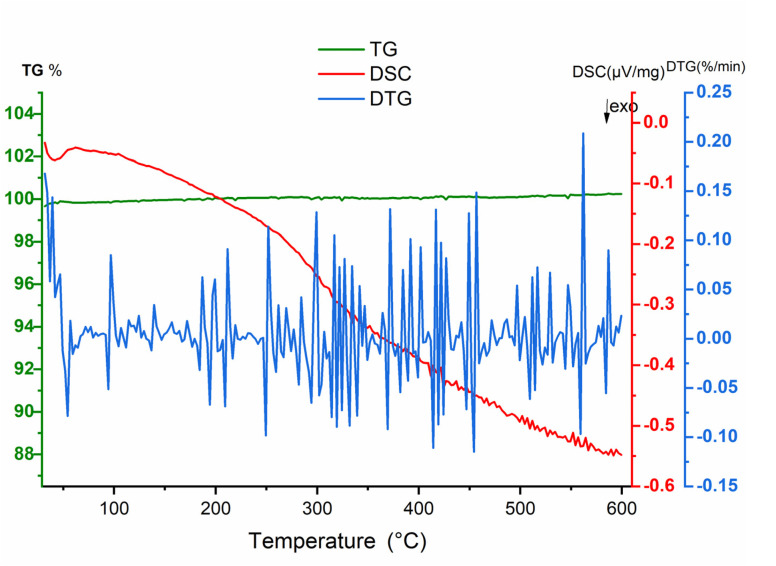
Thermal analysis results obtained from NdCaFeO_6_ ceramic. The thermogravimetric (TGA, green curve) signal shows flat curve indicating mass stability. Differential Scanning Calorimetry (DSC, red curve) displays uniform variation related to grain reordering *versus* temperature. The derivative thermogravimetry (DTG, blue curve) highlights almost weak variation of the mass.

Thermal analysis was performed under air. TGA curve did not indicate a clear weight lost characterized by the flat curve where the DTA curve showed no significative variation *versus* temperature (Fig. 15). The absence of a sharp pic on the DSC curve indicates also no phase transition in the material.

In the absence of a phase transition, these behaviors could be attributed to different electrical conductivity regimes within the material, influenced by both temperature and frequency.

#### 3.5.2. Impedance spectroscopy analysis

**Fig. 16:**
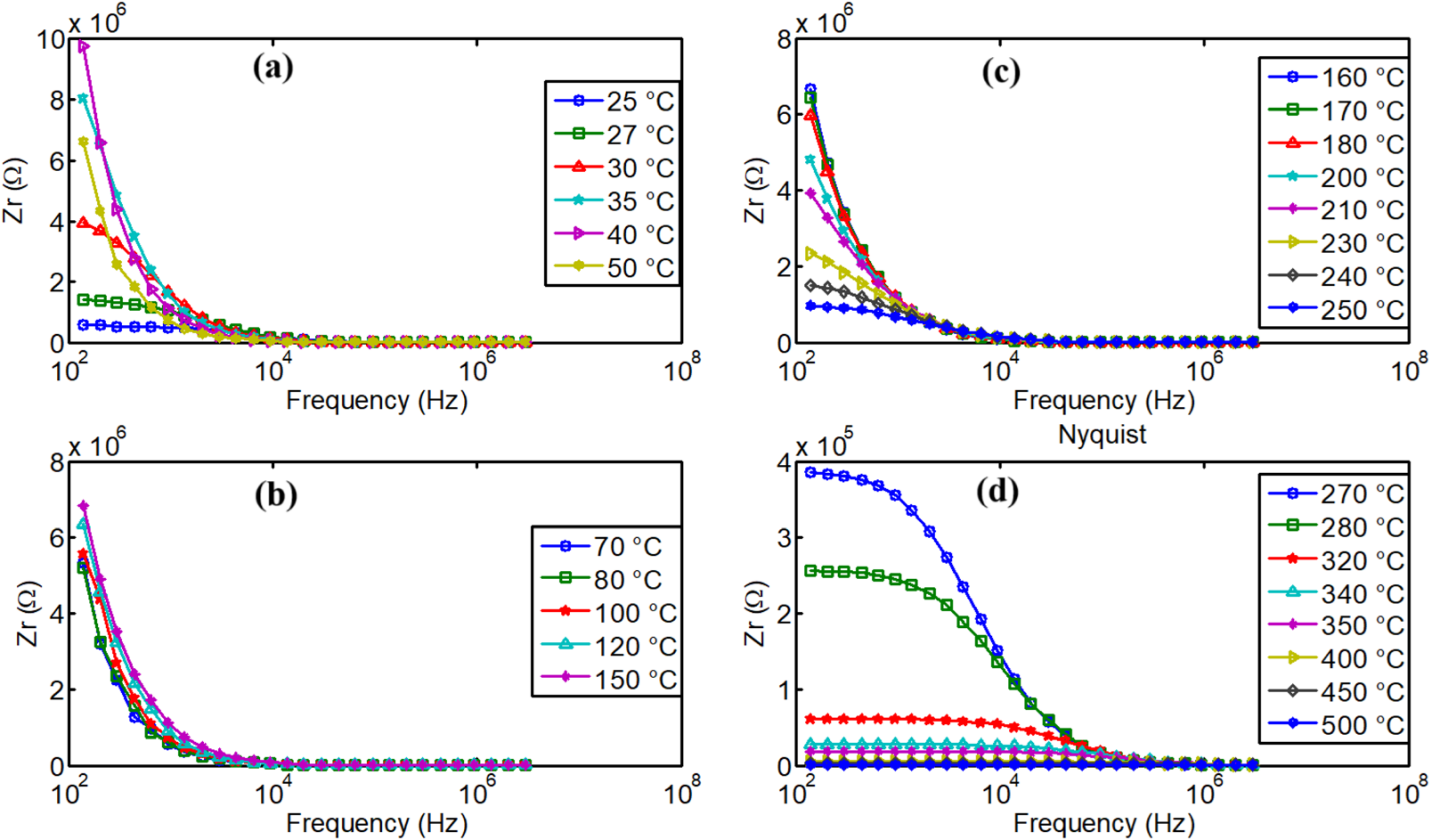
Impedance dispersion of real part of impedance in NdCaFeSnO_6_ ceramics.

The increase of NdCaFeSnO_6_ real impedance with temperature, in the range 25–70 °C, as observed in Fig. 16(a), is unexpected. This behavior may be attributed to several factors, including charge carrier trapping, grain boundary effects, oxygen ion dynamics and capacitance-related charge polarization as reported previously.^46,47^

At low temperatures, charge carriers may become trapped in localized states (*e.g.*, defects or impurities), temporarily increasing impedance until thermal activation enhances conduction. Additionally, slight temperature variations can modify defect distributions at grain boundaries initially, leading to higher resistance. In this temperature range an imbalance in oxygen ion mobility may further contribute to a transient increase in impedance before conductivity further improves. When increasing temperatures, charge polarization effects can intensify, that led to increase the resistive component of impedance before dominant conduction mechanisms take over.^48,49^

From 80 °C, we can remark the progressive decrease of real part of the impedance when temperature increases and the material exhibits normal electrical conductivity that increases with the temperature. This conductivity is governed by various mechanisms, including grain conductivity and grain boundary conductivity, facilitated for example, by hopping or oxygen vacancies.^50^Fig. 16(b–d) illustrate high dispersion of the material's real part of impedance, for several temperatures. Distinct relaxation frequencies were observed in the low frequency region in the temperature range 270–500 °C, that correspond also to high conduction region. Conduction mechanism is further analyzed using impedance spectroscopy data for a more comprehensive understanding.

**Fig. 17:**
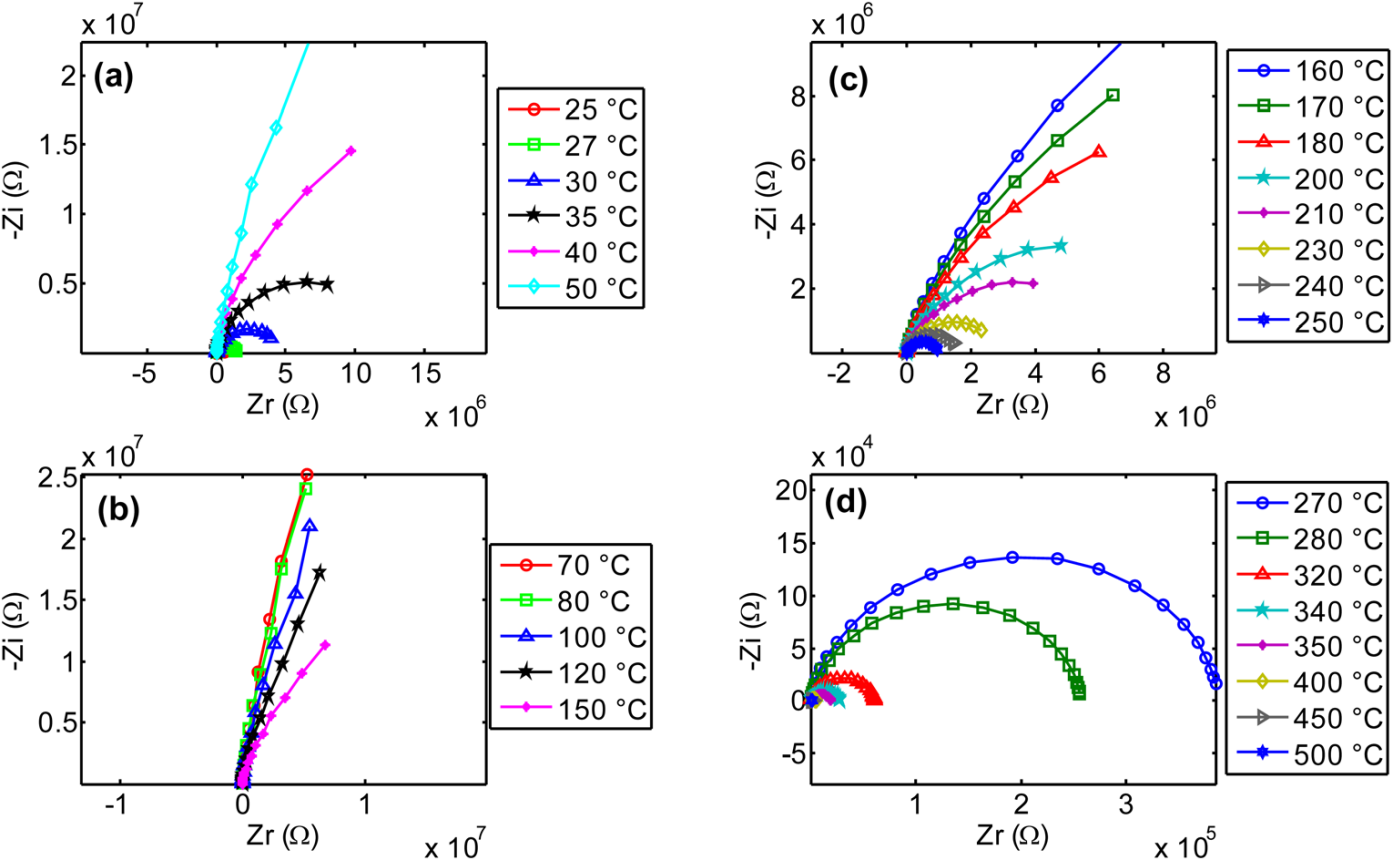
Nyquist plots of NdCaFeSnO_6_ impedance in several range of temperatures.

Fig. 17(a–d) presents the Nyquist diagrams across four different temperature ranges. At low temperatures (Fig. 17(a)), as expected, in agreement with previous observations, the radius of the semicircle increases abnormally with temperature. This unusual behavior was explained in previous paragraphs. In contrast, for temperatures above 80 °C (Fig. 17(b–d)), the opposite effect is observed: the radius of the imperfect semicircles decreases as temperature increases. This confirms the earlier findings regarding the activation of ionic conductivity in this compound, as well as the possible contribution of other charge transient mechanisms. It is important to note that the observed relaxation phenomena do not follow a pure Debye-type behavior due to the distribution of relaxation times and the complex nature of the conduction mechanism. The electrical properties of the bulk ceramic material can be distinguished from those of the grains, grain boundaries, and electrode contributions by employing frequency-dependent impedance spectroscopy at various temperatures. A clear reduction in grain and grain boundary resistance is observed, along with a negative temperature coefficient of resistance (NTCR) behavior, like that of semiconductors,^51,52^ characterized by imperfect semicircles in the Nyquist plots.

**Fig. 18:**
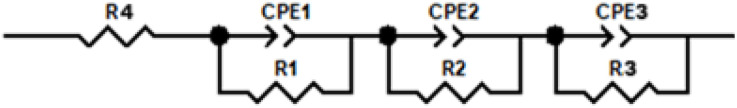
Equivalent electrical circuit (EEC) use to fit impedance data to determine conductivity mechanism in the material.

Using an equivalent circuit model, the experimental data (in Nyquist representation) were fitted, allowing us to determine the contributions of grain, grain boundary, and electrode effects to the overall dielectric response of the ceramic material. An equivalent circuit analysis (Randle circuit) was conducted to fit the Nyquist plots for each temperature. Among the multiple circuit configurations tested, satisfactory results were achieved with a model consisting of a series connection of three parallel R-CPE branches and a very low conducting resistance for full satisfactory results. The equivalent circuit diagram is presented below in Fig. 18.

**Fig. 19:**
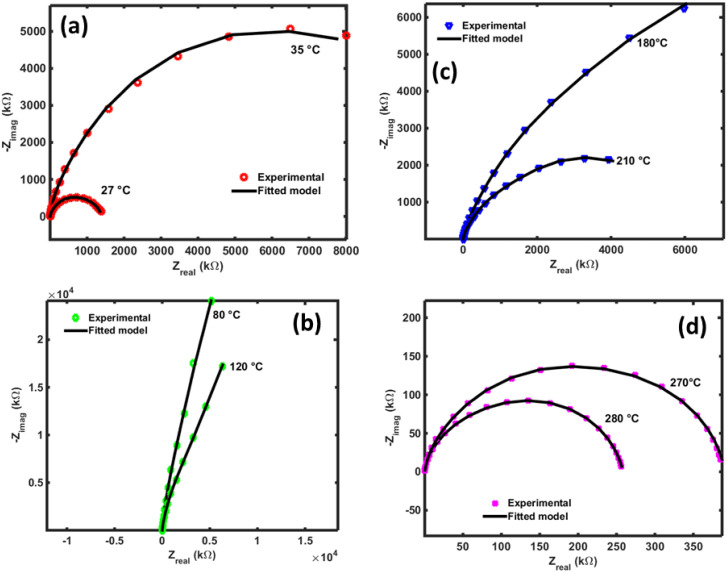
Experimental and fitted curves of NdCaFeSnO_6_ impedance spectral responses in Nyquist plots for some temperatures chosen in the 3 regions.

Recall that the CPE (Constant Phase Element) is a charge phase element characterized by an impedance *Z* given by the formula:
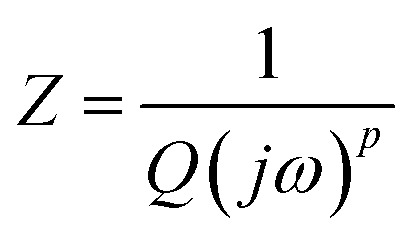


The constant phase element (CPE) corresponds to an ideal capacitor with *C* = *Q* for *p* = 1 and to an idealresistor with 
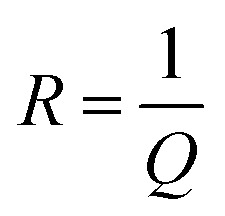
 for *p* = 0. Therefore, *p* can be used to represent the degree of imperfection of the capacitor, and represents a measure of arc distortion below the real impedance axis.^53^ The parameter *p* is related to the depression angle *α*_d_ given as following expression:
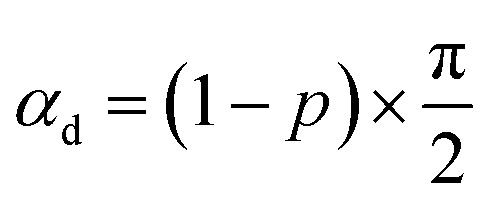


From this analysis, we determined the resistances of the grain (R3), grain boundary (R2), defects and space charge effects at electrodes (R3) and system wiring resistance (R4). Then, we calculated the conductivity through the material separating clearly the contribution of grain and grain boundary. We report on Fig. 19 (a–d) the experimental data and fitted curves for several temperatures chosen in between the previous defined temperature ranges.

Data are fitted satisfactorily in accordance of the Randle circuit shown in Fig. 19. The calculated values of the resistances of the materials at different temperatures are summarized in Table 6. In this table we can see clearly, the decrease of all the resistance when temperature increases. This is in favor of high conductivity at higher temperatures.

**Table 6:** Results of data fitted basing on Randle circuit in Fig. 19

*T* (°C)	*R*_3_ (Ω)	*Q* (F)	*p*	*R*_3_ (Ω)	*Q* (F)	*p*	*R*_3_ (Ω)	*Q* (F)	*p*	*R*_4_ (Ω)
27	6.67 × 10^5^	8.11 × 10^−10^	8.60 × 10^−1^	9.35 × 10^4^	2.13 × 10^−10^	9.28 × 10^−1^	6.32 × 10^5^	8.84 × 10^−11^	9.83 × 10^−1^	1.23 × 10^2^
30	1.32 × 10^6^	2.61 × 10^−10^	1.10 × 10	2.77 × 10^6^	7.45 × 10^−11^	9.72 × 10^−1^	2.59 × 10^5^	2.83 × 10^−10^	8.93 × 10^−1^	1.08 × 10^2^
35	8.39 × 10^6^	1.07 × 10^−10^	1.01 × 10	3.10 × 10^6^	9.51 × 10^−11^	9.81 × 10^−1^	4.47 × 10^5^	4.30 × 10^−10^	8.65 × 10^−1^	2.52 × 10^2^
70	2.84 × 10^8^	6.01 × 10^−11^	9.55 ×10^−1^	1.37 × 10^6^	8.00 × 10^−10^	9.41 ×10^−1^	2.79 × 10^5^	2.17 × 10^−9^	8.81 ×10^−1^	1.11 × 10^2^
80	2.99 × 10^8^	6.46 × 10^−11^	9.59 ×10^−1^	2.42 × 10^6^	4.86 × 10^−10^	9.04 ×10^−1^	2.76 × 10^5^	3.11 × 10^−10^	1.42 × 10	5.23 × 10^1^
120	1.35 × 10^8^	9.26 × 10^−11^	9.57 ×10^−1^	4.02 × 10^6^	2.17 × 10^−10^	9.23 ×1 0^−1^	7.72 × 10^4^	6.12 × 10^−10^	1.00 × 10	1.21 × 10^2^
180	1.54 × 10^7^	2.41 × 10^−10^	9.42 ×10^−1^	3.03 × 10^6^	2.30 × 10^−10^	9.27 × 10^−1^	4.76 × 10^5^	3.72 × 10^−10^	8.87 ×10^−1^	1.83 × 10^3^
210	4.59 × 10^6^	3.43 × 10^−10^	9.18 ×10^−1^	1.15 × 10^6^	1.88 × 10^−10^	9.43 ×10^−1^	1.65 × 10^5^	2.73 × 10^−10^	9.19 ×10^−1^	4.74 × 10^1^
250	3.69 × 10^7^	1.64 × 10^−10^	9.78 × 10^−1^	5.54 × 10^5^	8.16 × 10^−10^	8.90 × 10^−1^	4.10 × 10^5^	2.26 × 10^−10^	9.04 ×10^−1^	1.01 × 10^2^
270	1.65 × 10^5^	1.10 × 10^−9^	8.88 × 10^−1^	2.59 × 10^4^	3.00 × 10^−10^	9.32 × 10^−1^	2.00 × 10^5^	2.33 × 10^−10^	9.01 × 10^−1^	1.83 × 10^2^
320	4.11 × 10^3^	3.88 × 10^−11^	1.07 × 10	4.75 × 10^4^	2.86 × 10^−10^	8.85 × 10^−1^	8.93 × 10^3^	8.91 × 10^−9^	8.34 × 10^−1^	2.55 × 10
450	2.37 × 10^3^	2.94 × 10^−9^	7.27 × 10^−1^	4.65 × 10^2^	1.26 × 10^−4^	1.12 × 10^−2^	2.27 × 10^4^	1.21 × 10^−13^	4.24 × 10	3.70 × 10^2^
500	1.03 × 10^3^	5.57 × 10^−10^	8.82 × 10^−1^	1.39 × 10^2^	3.98 × 10^−5^	2.24 × 10^−1^	1.49 × 10^6^	4.24 × 10^−12^	7.38 × 10	4.88 × 10^2^

**Fig. 20:**
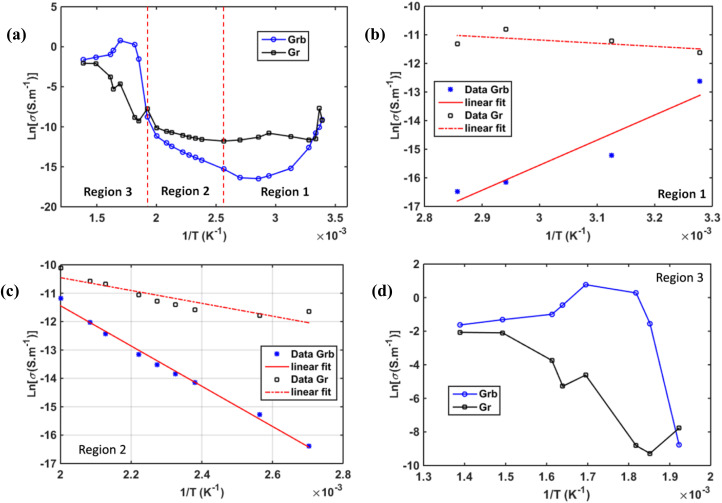
Arrhenius plot of conductivity based on impedance spectroscopy measurements: (a) global temperature range behavior. Conductivity curves in (b) Region 1 [30–70 °C], (c) Region 2 [80–250 °C] and (d) Region 3 [270–500 °C].

In Fig. 20, we report the results of the conductivity analysis for this material. Fig. 20(a) shows the overall temperature evolution of conductivity in this compound for the global temperature range. The conductivity evolution is quite different according to temperature ranges separated in three regions: Region 1 corresponds to [30–70 °C] (Fig. 20(b)), Region 2 to [80–250 °C] (Fig. 20(c)), and Region 3 to [270–500 °C] (Fig. 20(d)). As mentioned previously, the dynamics of conductivity in these separated regions are distinct. Only Region-2 can be described by a normal thermally activated ionic conductivity, which followed Arrhenius-type law^54^ with activation energies of 0.31 eV for the grains and 0.97 eV for the grain boundaries. The conductivity in Region 1 is unstable but can be described with Arrhenius plot with positive slop traducing the increase of resistivity that limits electrical conductivity. This phenomenon was attributed to atomic reordering and charge carrier trapping as described in previous paragraph. In Region 3, another mechanism was clearly observable that is different to that observed in region 1 and 2. This region corresponds to the high temperature range where electrical conductivity was maximal. The mechanism of conduction does not follow Arrhenius law. That is why to better understand the mechanism of conductivity we performed data adjustment using Jonscher type law in this region.^55^

**Fig. 21:**
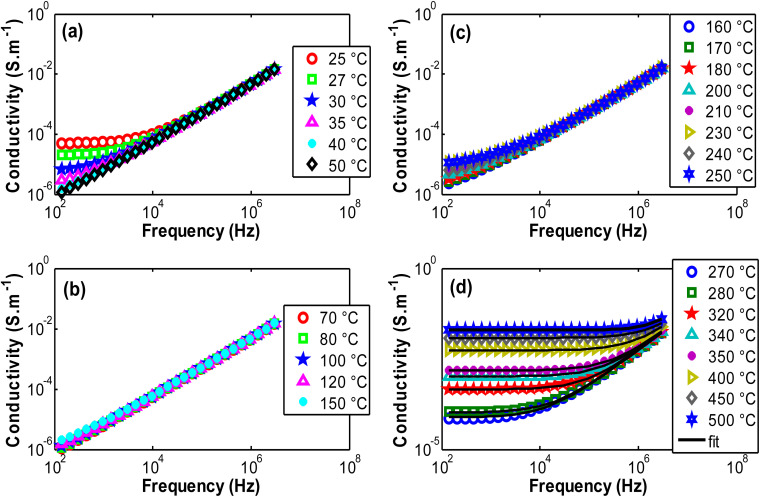
AC conductivity variation as function of frequency in several temperature regions. These curves were fitted with Jonscher law satisfactorily.

Fig. 21(a–d) illustrate the evolution of the ac conductivity calculated from impedance modulus as a function of temperature across the three regions. At low temperatures, a decrease in conductivity is observed with temperature, displaying a plateau-like behavior in the low-frequency region relative to DC contribution. This is followed by a positive slope at higher frequencies, beyond 10^4^ Hz, where the conductivity increases linearly, characteristic of the Jonscher effect.^55^ At intermediate temperatures (70–250 °C), a linear increase of the conductivity curves is observed for all frequencies (Fig. 21(b and c)) while a plateau began to be observed 250 °C, that was amplified by thermo-activated ionic conductivity, strongly dependent on the frequency at high temperatures. At high temperatures (270–500 °C), the impedance behavior exhibits clearly a low-frequency plateau (DC) followed by a linear increase at higher frequencies, with a more accelerated dynamic. The plateau enlarged more and more when temperature increased signature of DC predominance that can be attributed to electron appearance. This progression is observed in the correct temperature order, as shown in Fig. 21(d). This behavior is supported by Jonscher law described by the equation:^55^*σ*_ac_ = *σ*_dc_ + *Aω*^*α*^.

Basing on this expression, all conductivity curves were fitted to determine all the coefficients (*σ*_ac_: ac conductivity, *σ*_dc_: dc conductivity, *A*: the pre-exponential factor intrinsic to the material and *α*: exponent of the frequency, the Jonscher coefficient). The obtained values were summarized in Table 7 and some fitted curves were plotted on Fig. 21(d) in Region 3.

**Table 7:** Results of experimental data fitting by Jonscher law

Region-1				Region-2				Region-3			
*T* (°C)	*σ*_dc_ (µS m^−1^)	*A* (ng)	*α*	*T* (°C)	*σ*_dc_ (µS m^−1^)	*A* (ng)	*α*	*T* (°C)	*σ*_dc_ (mS m^−1^)	*A* (ng)	*α*
25	47.5	1.62	0.953	80	0.130	0.78	0.999	270	0.077	0.40	1.046
27	19.8	1.07	0.979	100	0.132	0.79	0.999	280	0.115	0.43	1.042
30	5.16	1.70	0.950	120	0.633	0.79	0.999	320	0.455	0.39	1.048
35	2.72	0.75	1.000	150	0.985	0.81	0.999	340	1.047	0.40	1.048
40	1.59	1.23	0.970	160	2.203	0.77	1.002	350	1.512	0.48	1.037
50	0.09	1.24	0.970	170	2.191	0.78	1.002	400	5.439	0.45	1.046
70	0.12	0.78	0.999	180	2.437	0.79	1.002	450	11.890	0.07	1.159
n = nano, *g* = [(S m^−1^) × (rad s^−1^)^−α^] is the pre-factor Aunity in the Jonscher law				200	3.121	2.86	0.923	500	19.730	0.01	1.263
				210	6.790	0.81	1.001				
				230	12.060	0.83	1.000				
				240	18.860	2.02	0.946				
				250	25.470	0.61	1.019				

The results in Table 7 showed the increase of DC conductivity as a function of temperature. Enlargement of DC current plateau is observed in Region 3, the high temperature region. In Region 1 the plateau is almost shrined when temperature increased by the charge trapping phenomenon, while intermediate temperature phase (Region 2) exhibited almost linear behavior. The fitted curves, using Jonscher law, are added Fig. 21(d) which demonstrates accurate results obtained in region 3. This figure showed more and more DC current creation when temperature increases.

#### 3.5.3. *I*–*V* measurements

To experimentally investigate the DC conductivity mechanism in NdCaFeSnO_6_ ceramics, a series of current–voltage (*I*–*V*) measurements were performed on a sample with systematically applied electric voltages, ranging from −8 V to 8 V, corresponding to an electric field from −0.150 kV cm^−1^ to 0.150 kV cm^−1^ within the sample.

**Fig. 22:**
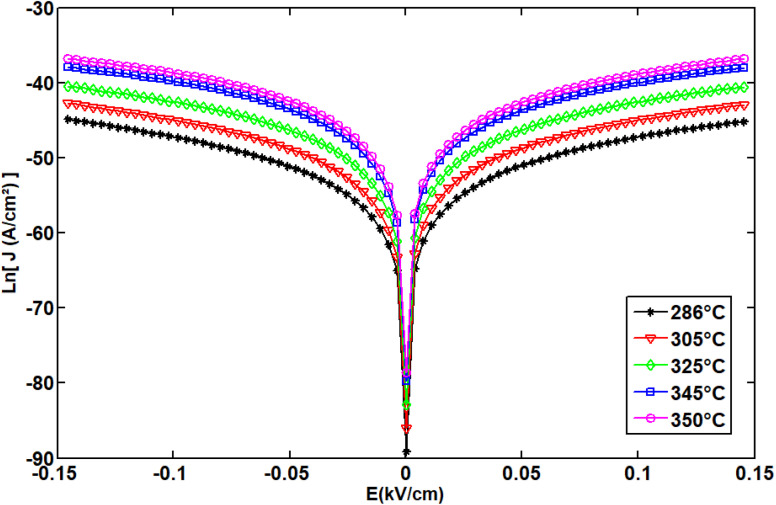
Diode like conduction mechanism in the NdCaFeSnO_6_ ceramic.

This method enables the identification of the field dependence of charge carrier transport across the compound. Additionally, to gain deeper insight into the underlying conduction mechanisms, *I*–*V* measurements were carried out at various temperatures. Analyzing the temperature dependence of the electrical response allows for the differentiation of transport phenomena such as thermally activated conduction, hopping mechanisms, defect-assisted charge movement, Schottky and Poole–Frenkel^50,55–58^ conduction, thereby providing a comprehensive understanding of the material's electrical properties. In the present case Poole–Frenkel conduction brought more accurate response of simulation particularly in the region 3 corresponding to the conductivity at high temperature range comparatively to those reported earlier in ref. 59, 60 and 61. Poole–Frenkel model was preferred after tested Schottky and Mott models.^62,63^Fig. 22 displays the evolution of current density as a function of the applied voltage in Region 3. A diode-like behavior is observed, with absence of hysteresis, indicating that there is no resistive switching effect and no rigid formation of a Schottky barrier at the interfaces.^64^ The threshold voltage is observed around ∼2 V, which corresponds to ∼0.036 kV cm^−1^ applied electric field.

It can be concluded that the conduction mechanism of the NdCaFeSnO_6_ compound follows a Poole–Frenkel (P–F) type mechanism, in Region 3 as the curve fitting results at high temperatures are satisfactory for the analysis of this material.^57,65^ Recall that the P–F effect describes the field-enhanced thermal ionization of trapped charge carriers in an insulating or semiconducting material. The characteristic equation for P–F conductivity is given by the emission current density, which can be expressed as follows:
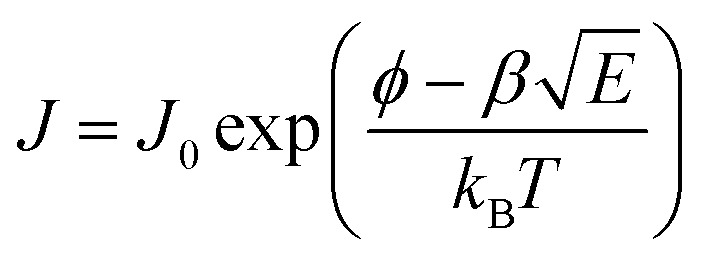
where, *J* is the current density, *J*_0_ is a prefatory, *ϕ* is the trap energy barrier without an applied field, *E* is the applied electric field, *β* is Poole–Frenkel coefficient, *k*_B_ is the Boltzmann constant, and *T* is the absolute temperature.

It is worth to recall that 
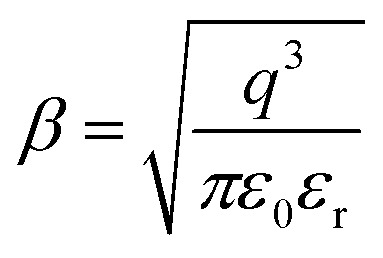
, where: *q* is the charge of electron (1.6 × 10^−19^ C), *ε*_0_ the vacuum permittivity (8.85 × 10^−12^ F m^−1^) and ε_r_the relative permittivity of the material.

To determine the activation energy of the NdCaFeSnO_6_ compound at high temperatures, we first plotted Ln (*J*) as a function of √*E*. The intercept values on the *y*-axis were then extracted for each temperature and subsequently plotted as a function of 1/*T*. The slope of the resulting linear fit enabled the calculation of the activation energy.

**Fig. 23:**
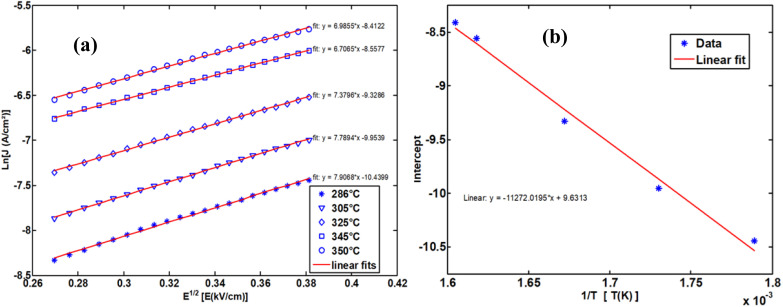
The Poole–Frenkel model employs a variety of representations to ascertain the activation energy.

The upper branches of these curves were modeled based on the Poole–Frenkel (P–F) relation, leading to the construction of Fig. 23(a), which illustrates the current density as a function of √*E*, exhibiting linear behavior. The linear fit of these curves provided the intercept on the *y*-axis, which, when plotted against 1/*T*, allowed for the determination of the activation energy in this temperature range.

The obtained activation energy was 1.55 eV, a relatively high value compared to that derived from impedance spectroscopy measurements. In the latter case, excitation in a frequency dynamic mode facilitates faster charge carrier motion, thereby reducing the activation energy. Fig. 23(a) presents the DC current density as a function of √*E*, while Fig. 23(b) depicts the variation of the intercept as a function of 1/*T*. The slope of this second plot was used to extract the P–F activation energy.

#### 3.5.4. Discussions

In this material, several mechanisms contribute to the electrical conductivity, including electronic, ionic, and space charge (defect) conductivity, each with complex conduction behaviors depending on frequency and the temperature regions. Firstly, at low temperatures (25–70 °C), atomic reorganization within the structure impedes the movement of charge carriers. As the temperature rises, conductivity activation begins gradually, following a Jonscher-type process. This results in a weak conduction zone for DC conductivity and a strong frequency dependence, confirming the presence of ionic AC conductivity. This is attributed to the valence change of Fe^3+^ to Fe^4+^ ions, depending on the temperature, which facilitates conduction *via* charge carrier hopping, as described by the Arrhenius law (in the range of 70–250 °C).

Finally, at higher temperatures (from 270–500 °C), a transition occurs, characterized by a conduction mechanism that follows Jonscher's law and exhibits a wide frequency range. In this high-temperature range, grain boundary conductivity surpasses that of the grains, with a crossing point observed around 280 °C. This transition occurs without a structural phase change, yet it reflects different thermoactive conduction regimes in the presence of the diffect chemical element such us Fe and Sn. Additionally, a Poole–Frenkel (P–F) type conduction mechanism dominates, that was evidenced by *I*–*V* measurements. Overall, this material demonstrates promising optoelectronic properties, making it suitable for various technologie applications.

## 4. Conclusion

The polycrystalline NdCaFeSnO_6_ powder was successfully synthesized *via* the solid-state method, and its structural and optoelectronic properties were extensively studied. The results revealed a complex arrangement of rare-earth, alkaline-earth, and metal oxides within a distorted double perovskite-related framework, contributing to a range of interesting physical properties, particularly in optical absorption and electrical conductivity across various temperature regions.

Structural analysis *via* Rietveld refinement of X-ray diffraction data confirmed a monoclinic crystal system with the *P*2_1_/*c* space group. Crystallographic analysis revealed nanocrystalline domains with sizes ranging from 30 to 90 nm, as determined by Scherrer and Williamson–Hall methods. SEM observations indicated larger particle sizes (226 nm) due to agglomeration, while SEM and EDS confirmed the material's crystallinity, morphological homogeneity, and elemental composition. These complementary techniques together provide a comprehensive understanding of the structural and chemical characteristics of the synthesized powder. Furthermore, Raman spectroscopy validated the XRD results, revealing the vibrational modes of Ca, Nd, Fe, Sn, and O ions and highlighting the connectivity of the (Sn/Fe)O_6_ octahedra in the monoclinic double perovskite structure. UV-visible spectrophotometry demonstrated strong optical absorption around 391 nm in the UV region, giving the sample a distinct green color. The determination of direct optical band gap yielded 2.44 eV value, notably lower than those reported for other calcium-stannate-based perovskites, establishing NdCaFeSnO_6_ as a promising low-bandgap semiconductor. The material's electrical properties were thoroughly analyzed with a focus on ionic conduction mechanisms. Impedance spectroscopy revealed charge carrier hopping at intermediate temperatures, with activation energies of 0.97 eV for grain boundaries and 0.31 eV for grains. Additionally, *I*–*V* measurements indicated a Poole–Frenkel conduction mechanism at higher temperatures, with an activation energy of 1.55 eV. The comparison of these conduction processes underscores the potential of NdCaFeSnO_6_ as a promising candidate for optoelectronic applications.

## Author contributions

Sondes CHAHLA: carried out the preparation of the material, collection and data analysis and paper draft. Yaovi GAGOU: participated in physical measurements, results interpretation, and manuscript revision, impedance spectroscopy analysis. Hanen CHAKER: was responsible for the methodology, data curation, writing, visualization, validation and supervision. Mimoun EL MARSSI and Rached Ben Hassen participates in XRD, Raman. All authors have read and approved the final version of the manuscript.

## Conflicts of interest

The author(s) declared no potential conflicts of interest with respect to the research, authorship, and/or publication of this article.

## Data Availability

This statement serves to certify that all authors affirm the availability of the data underpinning the findings of this study. These data may be obtained from the corresponding author upon justified request.
